# Breath-Figure Self-Assembly, a Versatile Method of Manufacturing Membranes and Porous Structures: Physical, Chemical and Technological Aspects

**DOI:** 10.3390/membranes7030045

**Published:** 2017-08-16

**Authors:** Edward Bormashenko

**Affiliations:** 1 Department of Chemical Engineering, Biotechnology and Materials, Engineering Faculty, Ariel University, P.O.B. 3, 407000 Ariel, Israel; Edward@ariel.ac.il; Tel.: +972-0747296863; 2 Tyumen State University, 6 Volodarsky St., Tyumen 625003, Russia

**Keywords:** membranes, polymer solution, breath-figures, ordering, capillary cluster

## Abstract

The review is devoted to the physical, chemical, and technological aspects of the breath-figure self-assembly process. The main stages of the process and impact of the polymer architecture and physical parameters of breath-figure self-assembly on the eventual pattern are covered. The review is focused on the hierarchy of spatial and temporal scales inherent to breath-figure self-assembly. Multi-scale patterns arising from the process are addressed. The characteristic spatial lateral scales of patterns vary from nanometers to dozens of micrometers. The temporal scale of the process spans from microseconds to seconds. The qualitative analysis performed in the paper demonstrates that the process is mainly governed by interfacial phenomena, whereas the impact of inertia and gravity are negligible. Characterization and applications of polymer films manufactured with breath-figure self-assembly are discussed.

## 1. Introduction

Well-defined micro- and nanoscaled porous polymeric architectures are in great demand for their use in advanced devices, including membranes [1,2,3,4], sensors [5,6], bio-engineering [7,8] and water-oil and size-selective separation processes [9,10,11,12,13]. One of the most versatile, simple, single-stage, and inexpensive methods, enabling manufacturing of porous polymer films with finely controlled topography, is so-called “breath-figure self-assembly” [14,15]. Historically the breath-figures method has been used since the 1850s by photographers as a simple and effective way to detect oil contamination on glass substrates [16]. An extended review of the numerous applications of the ordered, microporous films obtained with breath-figure self-assembly may be found in [16].

The formation of water droplets on solid surfaces was first investigated by Aitken in 1893 [17] and later in 1911 by Rayleigh [18,19]. The notion of “breath-figures” refers to the set of fog droplets that forms when water vapor comes into contact with a cold surface (solid or liquid). Breath-figures are a phenomenon commonly observed in daily life. One example is the surface fog that appears on a window when we breathe on it [20]. Another example is the formation of dew [21]. Knobler and Beysens investigated the formation of breath-figures under water droplet condensation [22,23,24,25] and found that they formed not only on solid surfaces but on liquids as well, specifically on paraffin oil [24]. The main features of the process of breath-figure self-assembly have been revealed through detailed study of water condensation [22,23,24,25], including the intriguing effect of non-coalescence of sessile droplets, to be discussed later. The interest in breath-figures self-assembly was revived when Widawski Francois and Pitois et al. demonstrated in a series of papers [26,27,28,29,30] that breath-figure self-assembly allows for the formation of microporous reliefs with well-controlled topography when polymer solutions are rapidly evaporated in a humid atmosphere. It should be emphasized that although the process looks simple, it involves almost all events inherent to interface science, namely: evaporation of a solvent, condensation of water droplets, instabilities developed in the evaporated polymer solution, delayed coalescence of closely packed droplets, and their interaction [16]. Thus, the process of breath-figure self-assembly may be understood only within the broad context of surface science [31,32,33,34].

## 2. Impact of the Polymer Architecture and Physical Parameters of the Process of Breath-Figure Self-Assembly

**Figure 1:**
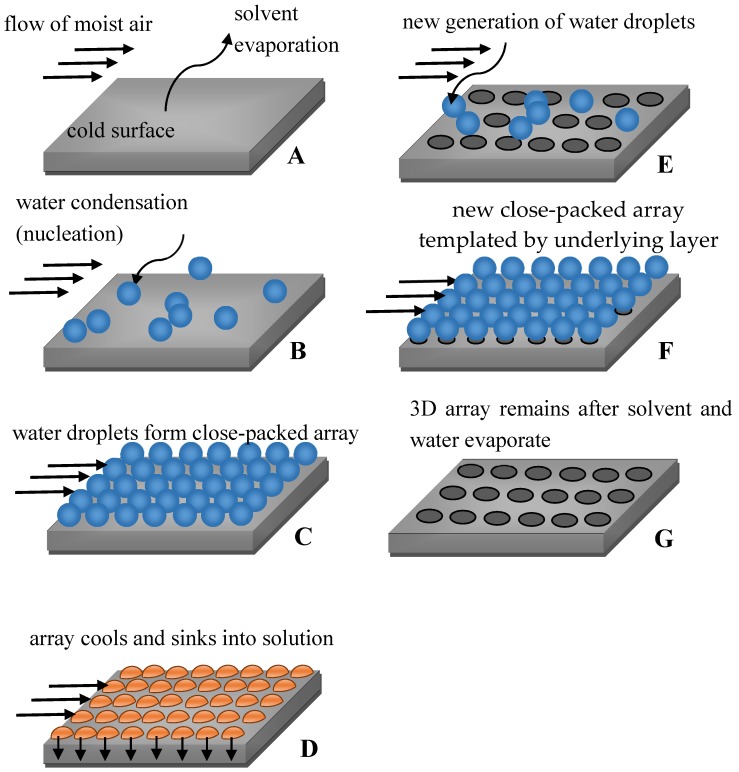
Sequence of stages resulting in breath-figure self-assembly. (**A**–**D**) formation of the first row of pores; (**E**–**G**) Formation of the second row of pores.

The details of breath-figure self-assembly remain mysterious, and no general mechanisms have adequately explained all experimental results [16]. It is agreed that rapid evaporation of the solvent cools the solution/humid air interface, resulting in intensive condensation of water droplets at the interface. The droplets then sink into the solution, eventually forming a honeycomb pattern, as depicted in Figure 1 [35,36].

However, results from different researchers often seem to conflict [16]. Consider first the impact of the polymer structure on the resulting pattern. Bolognesi et al. reported that polar groups of polymers play a fundamental role in the process [37]. These results were supported by findings reported in [38], where the authors reported that the hydrophilic end-groups can dramatically improve the film-forming property of polystyrene, and that the regularity of the film is mainly influenced by the interaction of film-forming polymers with condensed water droplets. Amirkhani et al. reported that the end-functional polymer produced a large area of regular spherical bubbles, whereas adding particles to the polymer solution leads to smaller arrays of flattened bottom bubbles. The separation length between pores was larger for the polymer/particle sample than for that of the end-functional polymer films [39].

**Figure 2:**
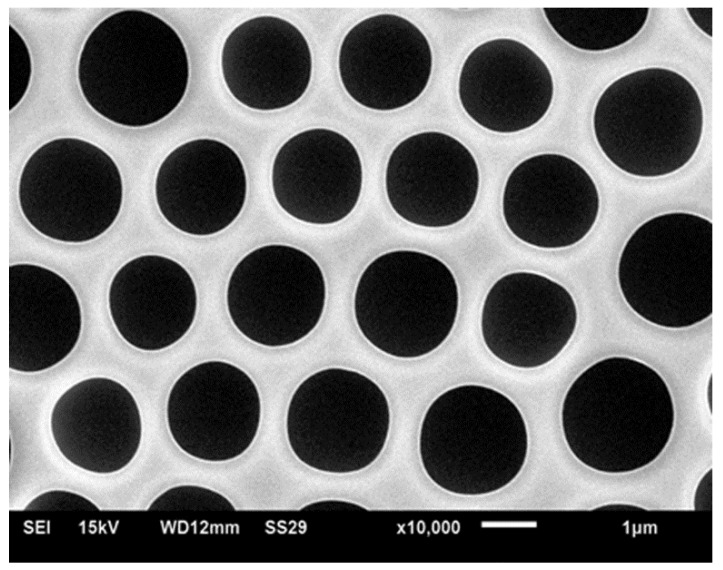
Typical honeycomb pattern arising from breath-figure self-assembly. The pattern was obtained by dip-coating the polyethylene substrate with the solution, containing 5 wt % of polycarbonate and a mixture of chlorinated solvents, namely: dichloromethane CH_2_Cl_2_ (90 wt %)/chloroform CHCl_3_ (5 wt %).

On the contrary, linear polystyrene without any polar end-group also led to ordered honeycomb structures when dissolved in toluene and CHCl_3_ solvents [40,41]. At first, it was suggested that star-like and hyperbranched polymers promote the formation of well-ordered structures [26,42]; however, later it was demonstrated that linear polymers also give rise to patterns typical of breath-figure self-assembly, such as depicted in Figure 2 [40,41]. It was suggested that coiled polymers (polystyrene) promote the formation of breath-figure patterns [42,43]; on the other hand, rigid-rod conjugated polymers also gave rise to well-ordered honeycomb reliefs [36]. The impact of the molecular weight of the polymer on the resulting pattern remains obscure, and the data reported by various groups are controversial [44,45,46]. The concentration of the solution definitely impacts the self-assembly process; however, its impact remains unclear [47]. The typical patterns for breath-figure self-assembly were obtained with amorphous [46] and crystalline polymers [48]. An additional difficulty in identifying the impact of the polymer architecture on the resulting pattern arises from the affinity of the processes of phase separation and breath-figure self-assembly, giving rise to similar eventual honeycomb patterns [49].

Breath-figure self-assembly has been carried out with thermoplastic [14,15,16,41,44,45,46] and cross-linked polymers [50,51,52]. Several groups reported breath-figure patterns obtained with high performance, engineering polymers, such as polyimide [53], polyetherimide [54], polysulfone [45,55], and silicon-containing graft copolymer poly(dimethylsiloxane)-graft-polyacrylates (PDMS-g-PAs) [56].

The impact of the solvent also remains unclear. It is generally agreed that the rate of a solvent evaporation and the associated concentration- and temperature-dependent properties of a polymer solution define to a large extent the topography of the honeycomb pattern arising from the breath-figures process [57,58]. Ferrari et al. noted that the thermodynamic affinity between polymer and solvent is a key parameter for breath-figure formation, along with other solvent characteristics such as water miscibility, boiling point, and enthalpy [41]. A model predicting the evaporation profile of the casting solution in this process for low concentrations of polymer was proposed in [59]. By adding a small amount of a surface active compound, it is possible to create ordered arrays from other solvents and, thus, markedly broaden the applicability of this patterning process [60]. It was recently demonstrated experimentally that a broad diversity of solvents, including acetone, dichloromethane, chloroform, carbon tetrachloride, tetrahydrofuran, toluene, xylenes, carbon disulfide, and N-N dimethylformamide, give rise to porous patterns when dissolved polystyrene and polycarbonate were evaporated in a humid atmosphere [61]. It was demonstrated that all solutions, when sufficiently pre-cooled, gave rise to typical “breath-figure” patterns [61]. Thus, the decisive factor affecting the formation of the breath-figures pattern turns out to be the temperature of the evaporated solution [16,61].

Hence, the impact of the substrate used for the breath-figure self-assembly may be decisive due to the fact that the substrate may serve as a thermal bath stabilizing the temperature of the evaporated polymer solution; the slide thickness is also shown to be a crucial parameter in this process [59,61,62,63]. However, the experimental data related to the impact of the substrate on the breath-figure self-assembly remain scarce [42,61,62,64,65]. Valiayaveettil and co-workers used clean glass, epoxy, amine-terminated, and dendrimer-functionalized glass as well as silicon wafers to cast a poly(p-phenylene) with pyridine chloroform solution [65]. In this work, honeycomb membranes were obtained with glass and silicon wafers. In contrast, ring patterning, low pore density, or net-type structures were obtained from the epoxy-treated, amine-terminated, and dendrimer-functionalized glasses, respectively [65].

## 3. Processes Used for Breath-Figure Self-Assembly

Various experimental techniques were successfully applied for breath-figure self-assembly, including drop-casting [66,67,68,69,70,71], spin-coating [72,73,74,75,76,77,78], the “doctor-blade” technique [79,80], and dip-coating [46,54,58,81,82,83]. When breath-figures-inspired patterns are formed under drop-casting, a drop of polymer solution is deposited by a precise micro-syringe (or micro-injector) on a solid substrate and exposed to air flow with a controlled humidity [66,67,68,69,70,71]. The spin-coating process involves putting a polymer solution on the center of the substrate, which is either spinning at low speed or not spinning at all. The substrate is then rotated at high speed in order to spread the evaporated polymer solution by centrifugal force. The whole process is performed under controlled humidity [72,73,74,75,76,77,78]. In the doctor-blade technique, an immobilized blade applies a unidirectional shear force to the polymer solution that passes through a small gap between the blade and the substrate [79,80,81]. When honeycomb patterns are obtained with dip-coating, the solid substrate is pulled with a constant speed from the evaporated polymer solutions [46,54,58,82,83,84]. In all aforementioned processes, the impact of air humidity and the physico-chemical properties of a solid substrate may be decisive in constituting the topography of the resulting honeycomb relief [14,15,16,59,64,67].

## 4. Main Stages of Breath-Figure Self-Assembly

The main stages of breath-figure self-assembly are: nucleation of water droplets, condensation on the polymer solution/vapor interface, interaction between droplets, and final removal of water through its evaporation. Condensation is the formation of a liquid phase from the gaseous (vapor) one, which starts with “nucleation”. Nucleation is the formation of an embryo or nucleus of a new phase in another phase [32,85]. Homogeneous and heterogeneous nucleation scenarios should be distinguished. Heterogeneous nucleation takes place in the presence of foreign particles or surfaces, whereas homogeneous nucleation occurs while growing small clusters of molecules [86]. If it is thermodynamically favorable for these clusters to grow until they become recognizable droplets of the liquid phase [32,85,86]. It should be emphasized that the precise mechanism of nucleation during the breath-figure self-assembly remains unclear, due to the fact that the nuclei of water droplets are formed in the presence of a solvent vapor [87]. The processes of nucleation and condensation have a decisive influence on the formation of the eventual breath-figures-inspired pattern [87]; hence, additional experimental and theoretical efforts are necessary for elucidating the kinetics of nucleation and condensation taking place under the conditions of the breath-figure self-assembly.

It is also possible that nucleation occurs at the polymer solution/vapor interface. In this case the nucleation rate I (for its accurate definition, see [32,88,89,90,91]) is modified through the function depending strongly on the equilibrium (Young) contact angle $\theta_{Y}$, namely: $I\approx \exp\left(-\frac{\Delta G_{\max}^{het}(\theta_{Y})}{k_{B}T}\right)$, where $\Delta G_{\max}^{het}$ is the value of the potential barrier to be surmounted for heterogeneous nucleation, *T* is the temperature, and k_B_ is the Boltzmann constant. The value of $\Delta G_{\max}^{het}$ for a contact angle hysteresis-free substrate is given by:

(1) $$\Delta G_{\max}^{het}(\theta_{Y})=\Delta G_{\max}^{\mathrm{hom}}\frac{(2+\cos\theta_{Y}){\left(1-\cos\theta_{Y}\right)}^{2}}{4}$$

where $\Delta G_{\max}^{\mathrm{hom}}$ is the potential barrier of the homogeneous nucleation supplied by Equation (2):

(2) $$\Delta G_{\max}^{\mathrm{hom}}=\frac{\gamma 4\pi r_{c}^{2}}{3}$$

where $r_{c}$ is the size of the critical nucleus and *γ* is the surface tension [86,88].

Beysens et al. studied the formation of breath-figure patterns formed on cold borosilicate substrates, either pristine or hydrophobized by a solution of octadecyltrichlorosilane [22]. The treatment by octadecyltrichlorosilane enabled control of the apparent contact angle of the cooled solid substrate [22]. The pattern of water on glass was studied by direct observation and light scattering as a function of the contact angle $\theta_{Y}$, the velocity of vapor volume transfer “flux”, denoted as $\Phi_{vol}$, the degree of supersaturation ΔT, and time *t*. It was established that when $\theta_{Y}=0^{0}$, a uniform water layer forms whose thickness grows as *t* increases at a constant $\Phi_{vol}$ and ΔT. For $\theta_{Y}=0^{0}$, droplets are formed at a constant $\Phi_{vol}$ and ΔT; the radius of an isolated droplet grows as $t^{0.23}$, but as a result of coa1escences the average droplet radius grows as ~$t^{0.75}$ [22]. The most important conclusion following from these considerations is expected, namely that the eventual “breath-figure” pattern depends on the apparent contact angle $\theta_{Y}$, as predicted by Equations (1) and (2). The growth process is accompanied by coalescence of droplets and turns out to be similar; coalescences simply rescaled the distances and left the basic droplet pattern unaltered [22]. The details of the coalescence were addressed in [23]; the authors showed that the number of coalescences undergone by a given droplet grows logarithmically with time; the total distance traveled by this droplet is proportional to its size [23]. The experiments, reported by Beysens et al., supported the important information about the kinetics of formation of the “breath-figure” patterns [22,23]. However, these experiments were carried out under model conditions in the absence of evaporated polymer solutions, which essentially complicates the physics of the process; hence, novel experimental data shedding light on the kinetics of formation of breath-figure patterns occurring at the polymer solution/vapor interface are necessary.

## 5. Multi-Scale Patterning Observed under “Breath-Figure Self-Assembly”

**Figure 3:**
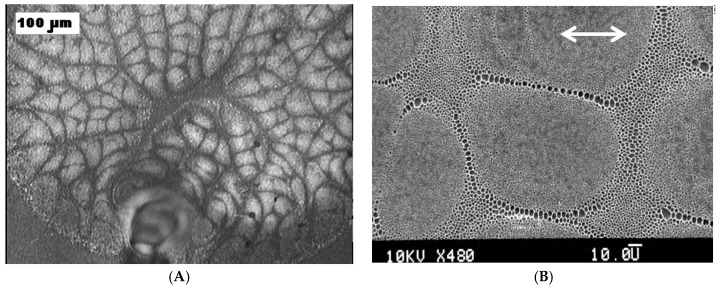
Large-scale pattern typical for breath-figure self-assembly (Polystyrene (5 wt %) was dissolved in a mixture of dichloromethane CH_2_Cl_2_ (90 wt %) and chloroform CHCl_3_ (5 wt %) and deposited by dip-coating on the polyethylene substrate). (**A**) The scale bar is 100 µm; (**B**) the scale bar is 50 µm [97] (Copyright 2007 Wiley).

Multi-scale, hierarchical patterning is featured in breath-figures [14,76,77,92,93,94]. The characteristic spatial scales of patterns vary from nanometers to dozens of micrometers. Consider the large-scale patterning observed in rapidly evaporated polymer solutions [94,95,96,97,98,99], depicted in Figure 3, observed during the dip-coating of substrates with rapidly evaporated polymer solutions (similar large-scale patterns were also observed under other experimental techniques [96,97,98,99]). In particular, it was demonstrated that the characteristic dimensions of cells constituting the pattern grow with the concentration of the polymer solution [95].

The physical mechanism of the patterning remains debatable. For film thickness less than about 100 nm (thin layers), effective molecular interactions between the liquid layer surface and the substrate dominate all other forces (like thermo- or soluto-capillarity or gravity) and thus determine the film stability and patterning under dewetting [100,101,102].

For evaporated films with a thickness above 100 nm, thermo-capillarity forces become dominant, resulting in instability caused by the Marangoni convection, either thermo- or soluto-capillary [103,104,105,106,107,108,109,110,111,112,113]. It should be mentioned that the analysis of the pattern inspired by thermo- and soluto-capillarity is an essentially non-linear one and involves complicated mathematical models [103,104,105,106,107,108]. It was demonstrated experimentally that the patterns observed in the evaporated (cooled from above) layers are different from those formed in a layer heated from below without evaporation [109]. Thus, the kinetics of evaporation, studied in [110], turns out to be crucial for constituting large-scale patterns. An apparatus utilizing self-organized liquid flow for the targeted modification of macromolecular systems in a solution was suggested in [112]. Marangoni-flow-induced patterns, observed under spin-coating of evaporated polymer solutions, were reported in [114]. When the polymer solution is evaporated, thermo- and soluto-capillary flows occur and both may contribute to the eventual pattern. It remains debatable what kind of Marangoni flow has a decisive impact on the pattern. The experimental data reported in [95,115] indicate that it is the solutal Marangoni flow that causes the pattern. Indeed, heating the substrate from below destroyed the pattern [115]. This contradicts the idea that self-organization is due to the jump in surface tension caused by a temperature gradient (temperature-gradient-driven Marangoni instability).

However, when evaporation is present, thermo- and soluto-capillarity represent only a few of a diversity of destabilizing mechanisms: vapor recoil, differential evaporation (the dependence of the evaporation rate on the thickness of the film), or, sometimes both contribute to the development of interfacial instability and as a result exert an influence on the pattern’s makeup [116]. de Gennes proposed an alternate mechanism of patterning. He showed that in an evaporating film, a “plume” of solvent-rich fluid induces a local depression in surface tension, and the surface forces tend to strengthen the plume. His calculations led to the conclusion that this kind of instability should dominate over the classic Bénard–Marangoni instabilities [117,118]. It should be emphasized that in all the aforementioned instabilities (namely Marangoni and de Gennes) the tangential vector field of velocities drives the liquid. This makes possible the topologically-based approach to patterning, as demonstrated in [119]. The “hairy ball theorem” of algebraic topology states that any continuous tangent vector film on a surface topologically equivalent to a sphere must have at least one point where the vector is zero [120]. Remarkably, the “hairy ball theorem” predicts the existence of at least one zero tangential velocity point at the surface of the evaporated polymer solution [119]. Indeed, these zero velocity points were observed experimentally [119].

The role of water droplets in the formation of large-scale patterns is shown in Figure 3. It seems that water droplets work as “tracers”, enabling visualization of the boundaries and separating the large-scale cells depicted in Figure 3. Pores appearing after the droplets’ evaporation are accumulated in zero-velocity points, demonstrating the “hairy ball theorem”, as discussed in [119,120,121].

Now consider the mesoscopic, micro-scaled patterning observed by various groups [14,15,16,26,27,28,29,30,35,36,53,67,68]. These mesoscopic patterns are built from well-ordered micro-scaled or sub-micro-scaled pores, demonstrating long-range 2D and sometimes 3D ordering [35,122,123,124]. The mechanism of this patterning was discussed in [48,66]. Govor et al. related the mesoscopic ordering to capillary interaction between droplets, discussed in detail in [125,126,127,128]. Kralchevsky et al. demonstrated that between two particles placed on the liquid/vapor interface a force (which may be either attractive or repulsive) similar to the Coulomb interaction acts between two endless wires charged with constant linear charge densities [125,126,127,128]. It is reasonable to suggest that this capillary interaction between droplets, condensed at the polymer solution/humid vapor interface, is responsible for the long-range ordering inherent to breath-figure self-assembly. It was already demonstrated by Bragg, Nye, and Lomer (in [129,130]), that the capillary interaction of bubbles promotes the assemblage of bubbles, representing the crystal structure of real metals.

**Figure 4:**
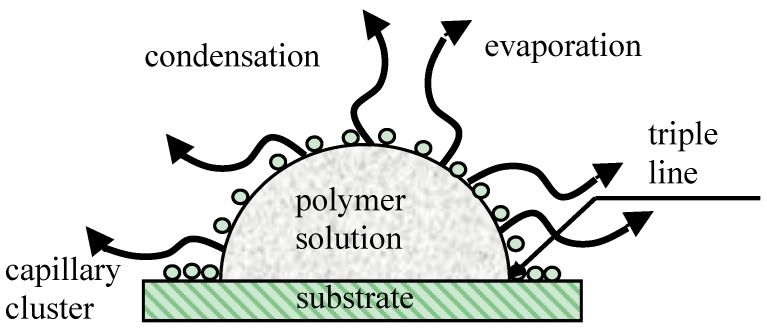
Breath-figure self-assembly taking place under drop casting is depicted. A droplet of the polymer solution is evaporated in the humid atmosphere. Water droplets are condensed at the polymer solution/vapor interface. A capillary cluster built from water droplets is formed in the vicinity of the triple (three-phase) line.

Capillary interaction is not the only kind of physical interaction between particles placed at the liquid/vapor interface. It was demonstrated by Pieranski that electrostatic interactions between floating particles may be no less important than capillary ones [131]. The role of the Marangoni thermo-capillary convection in the formation of ordered honeycomb patterns was mentioned in [66,132,133]. The details of the physical mechanism giving rise to the long-range self-assembly of pores, inherent to breath-figure patterning, remain unclear. It is noteworthy that the formation of ordered ensembles of droplets under the drop casting method occurs in the vicinity of the triple (three-phase) line, as shown in Figure 4 [134]. Self-assembly of colloidal particles (not droplets!) taking place in the vicinity of the triple line was studied extensively in [135,136]. However, the approach developed in [135,136] could hardly be extended to the explanation of the self-assembly of condensed water droplets, due to their coalescence (to be discussed below).

It was also demonstrated that some additives such as PEG and dendrons promote the ordering occurring under breath-figure self-assembly [62,137,138]. The well-ordered honeycomb patterns resulting from breath-figure self-assembly are evidence of non-coalescence or delayed coalescence of sessile water droplets condensed on the polymer solution/vapor interface. This means that a so-called “capillary cluster” built from micro-scaled water droplets exists on the polymer solution/vapor interface. The physical behavior of non-coalescent capillary clusters, in which capillary interactions prevail or play an essential role, has drawn the attention of investigators recently [139,140,141].

**Figure 5:**
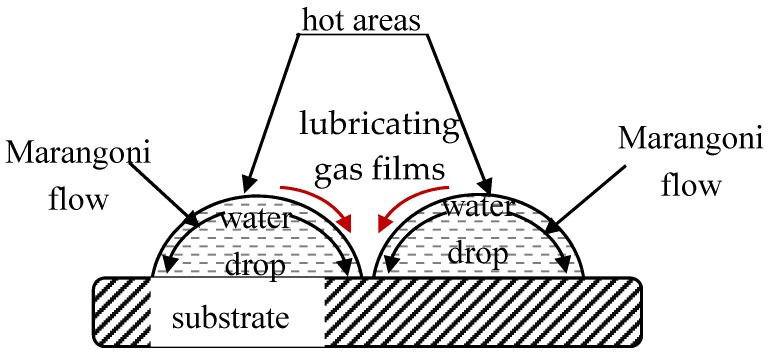
Scheme of non-coalescence of sessile droplets is depicted (see [147,148] for details).

When droplets of the same liquid touch one another, one expects coalescence [23,142,143]. The mechanism of the non-coalescence observed in capillary clusters remains disputable. Karpitschka et al. showed that sessile droplets from different but completely miscible liquids do not always coalesce instantaneously upon contact: the drop bodies remain separated in a temporary state of non-coalescence, connected through a thin liquid bridge [144,145,146]. Karpitschka et al. suggested that the delay originates from Marangoni convection [144,145,146]. Systematic study of Marangoni-convection-inspired non-coalescence was undertaken by Dell’Aversana et al. ([147,148]), who performed both laboratory experiments and molecular dynamics simulations. In the case of a pair of sessile droplets, a locally hotter region is formed in the center at the top of droplet, as takes place under the coffee-stain effect [149,150,151,152]. These surface-temperature variations not only give rise to thermo-capillary Marangoni convection within the droplets, as depicted in Figure 5, but also may drag air surrounding the drops into the space between them. This gas film serves to lubricate the space between the liquid surfaces, preventing them from coming into contact [147,148]. It is noteworthy that under breath-figure self-assembly the experimental situation is essentially complicated by the fact that sessile water droplets are located at the rapidly evaporated polymer solution/vapor interface. This may strengthen thermo-capillary Marangoni flows [151,152]. However, as will be demonstrated below in Section 6, the condensed droplets relatively rapidly come to thermal equilibrium; thus, the true role of the thermo-capillary Marangoni flows in preventing coalescence remains unclear. Other mechanisms of non-coalescence were discussed in [148]. We conclude that the details of the non-coalescence of droplets in capillary clusters remain unclear and call for further experimental and theoretical insights.

Nanoparticles also prevent the coalescence of droplets and enable the formation of the additional nanoscale in the hierarchical topographies obtained under breath-figure self-assembly (see the extended review of the state of the art of nanoparticles in breath-figure self-assembly in [16,153,154,155,156]. Saunders et al. demonstrated that the superlattice of mono-dispersed gold nanocrystals formed under the breath-figures process had an ordered structure at the nanometer scale [156]. The interaction between self-organization processes at the nano- and micrometer length scale, especially through the formation of a water droplets/evaporating polymer solution interface and droplets’ collective motions, was addressed in [157].

## 6. Main Physical Processes Involved in Breath-Figure Self-Assembly and the Hierarchy of Their Temporal Scales

Now consider the dimensionless numbers describing breath-figure self-assembly, namely the Bond (*Bo*), capillary (*Ca*) and Reynolds (*Re*) numbers:

(3) Bo=ρgL2γ, Ca=ηvγ, Re=ρvLγ

where *ρ* is the density (the densities of water and polymer solutions are very close), *η* and *γ* are the viscosity and surface tension of the polymer solution respectively, *v* is the characteristic velocity of droplets and pores, and *L* is the characteristic spatial scale.

Assuming for numerical values of physical parameters appearing in Equation (3):

$$\rho ≅1.0\times 10^{3}\frac{kg}{m^{3}};\gamma ≅25\times 10^{-3}\frac{J}{m^{2}};\eta ≅10^{-2}-10^{-1}Pa\times s;v≅10-30\frac{\mu m}{S}$$

(the viscosity of the solution is taken for the initial stage of the evaporation, and the velocity equals the maximal velocity of pores, established experimentally in [121]), we conclude that inequalities

(4) Bo<<1, Ca<<1

take place for all lateral characteristic scale lengths inherent to breath-figure self-assembly, namely: $10^{-9}m<L<10^{-5}m$. This means that the effects due to gravity, inertia, and viscosity are negligible, and breath-figure self-assembly is mainly driven by interfacial phenomena. However, the viscosity, growing with the evaporation of the polymer solution layer, helps to stabilize the eventual honeycomb pattern, as will be shown below. Indeed, the breath-figure patterns were observed on horizontal [14,15,16] and vertical [46,95,96,97] substrates. Moreover, multi-layered porous structures were observed for vertically driven substrates under the dip-coating process, evidencing the crucial role of interfacial processes, and the negligibility of gravity for the breath-figures process [62]. It is seen from estimations supplied by Equation (4) that breath-figure self-assembly is a “slow” process in which effects due to inertia and viscosity are negligible.

In order to clarify the precise meaning of the wording “slow process”, it is instructive to elucidate the hierarchy of time scales inherent to the process, namely: $\tau_{therm}^{sol}$ and $\tau_{therm}^{drop}$, which are the characteristic times necessary for attaining thermal equilibrium in the evaporated polymer solution and condensed water droplets, respectively; $\tau_{\mathrm{int}erf/visc}^{}$, which is the characteristic time necessary for viscous stresses (developed in the evaporated polymer solution layer) for balancing interfacial ones (at the length scale of a single droplet *R*), and eventually the temporal scales $\tau_{ev}^{sol}≅10s$ and $\tau_{ev}^{drop}≅1s$, which are the characteristic times of evaporation of the polymer solution and water droplets, respectively. The estimations for the time scales yield:

(5) $$\tau_{therm}^{sol}≅\frac{L_{sol}^{2}}{\alpha_{sol}};\tau_{therm}^{drop}≅\frac{R^{2}}{\alpha_{w}};\tau_{\mathrm{int}erf/visc}≅\frac{\eta R}{\gamma },$$

where $\alpha_{sol}$ and $\alpha_{w}$ are the thermal diffusivities of a polymer solution and water, respectively; $L_{sol}$ and *R* are the characteristic spatial scales of the evaporated layer of the polymer solution (in other words its typical thickness) and the condensed droplet (i.e., its radius), respectively; and *η* and *γ* are the viscosity and the surface tension of the polymer solution at the initial stage of the evaporation, as noted above.

Assuming: $\alpha_{sol}≅7.7\times 10^{-8}\frac{m^{2}}{s}$(as it is taken for the chloroform, as a typical solvent); $\alpha_{w}≅1.47\times 10^{-7}\frac{m^{2}}{s}$, $L_{sol}≅10^{-4}m,\;R≅10^{-6}m$, yields following rough estimations:$\tau_{therm}^{sol}≅0.13s;\tau_{therm}^{drop}≅7\times 10^{-6}s;\tau_{\mathrm{int}erf/visc}≅0.5\times (10^{-5}-10^{-6})s$.

Finally, we estimate the hierarchy of time scales inherent for the breath-figure self-assembly:

(6) $$\tau_{ev}^{sol}>\tau_{ev}^{drop}\geq \tau_{therm}^{sol}>>\tau_{therm}^{drop}\geq \tau_{\mathrm{int}erf/visc}$$

This hierarchy may be interpreted as follows: the condensed water droplet almost immediately comes to thermal equilibrium, whereas the polymer solution layer, fixing the pattern, is far from thermal equilibrium on the time scale of its evaporation. Equation (6) also implies that at the lateral scale of a single droplet, interfacial stresses are immediately balanced by viscous ones, which are developed in the polymer solution layer. So, the behavior of a single droplet is quasi-static, and the formation of pores is stabilized by the viscosity of the polymer solution layer, growing with the evaporation, whereas the dynamics and thermodynamics of large (~10 μm) cells, depicted in Figure 3, are essentially at non-equilibrium.

## 7. Characterization of Patterns Obtained with Breath-Figure Self-Assembly

### 7.1. Characterization of the Ordering of Patterns

Ordered honeycomb structures arise from breath-figure self-assembly. How may the 2D ordering of patterns be quantified? Two approaches to the quantifying of surface ordering have been reported. The first exploited the statistical properties of the autocorrelation functions [158], calculated over the pixels of SEM images taken of honeycomb patterns [62]. The correlational analysis of the SEM images indicated short-range and mesoscopic ordering of the honeycomb structures on the characteristic scale of 5 μm for the typical, weakly ordered patterns, represented in Figure 2. The second approach to the problem involved the use of Voronoi diagrams (or Voronoi tessellations), described in detail in [159,160]. Perfect ordering of pores on the scale of 1–10 mm was registered [14,15,16]. Voronoi tessellation and Voronoi entropy were successfully applied for the quantitative characterization of the ordering of capillary clusters (see [141]) and the pores constituting honeycomb reliefs in [73,161,162]. The study of defects (including line defects), observed under breath-figure pattering and possibilities of their elimination was reported in [163].

### 7.2. Surface Characterization of Patterns Obtained with Breath-Figure Self-Assembly

The simplest (and an extremely useful) method of characterization of micro-rough surfaces is based on the measurement of the apparent contact angle [32,33,34,164]. A detailed study of the apparent contact angles inherent for honeycomb, porous surfaces arising from breath-figure self-assembly was reported in [165,166,167]. It was demonstrated that honeycomb surfaces produced by breath-figure self-assembly demonstrate a pronounced heterogeneous (air-trapping) Cassie–Baxter wetting regime [79,168,169,170]. The interfaces characterized by stable Cassie–Baxter wetting have high apparent contact angles and low contact angle hysteresis [32,33,34,168,169,170]. Thus, they are suitable for a broad range of applications, where self-cleaning properties of the interface are demanded [171,172]. Somewhat surprisingly, polymer surfaces manufactured by breath-figure self-assembly showed the Cassie-like wetting, even when hydrophilized by a metal coating [167]. The mechanism of stability of the Cassie wetting states observed on honeycomb polymer and metallized reliefs was addressed in [173].

At the same time, the mechanical properties of microporous films obtained with the breath-figure self-assembly remain obscure. The non-trivial mechanical properties of these films are expected, owing to their reduced size and dimensionality compared with those of their macroscopic counterparts [174].

## 8. Novel Applications of Breath-Figure Self-Assembly

We already considered the numerous applications of interfacial honeycomb structures arising from breath-figure self-assembly in Section 1 (see also the comprehensive review in [16]). Some very recent applications of these structures are noteworthy. Microporous functional polymer surfaces arising from breath-figure self-assembly have been proven to be selective surfaces for attracting eukaryotic cells while maintaining antifouling properties against bacteria [175]. Polymer scaffolds prepared by the breath-figure technique enabled differentiation of human mesenchymal stem cells, as demonstrated in [176].

A process of manufacturing microspherical particles with the breathfigures process was reported in [177]. Breath-figure self-assembly was successfully employed in manufacturing solid-state supercapacitors [178]. It was shown that honeycomb polymer films supported by steel meshes enable the manufacturing of electrically controlled membranes [179]. Low friction and bubble-repellent surfaces with micro-dimple arrays, obtained with the breath-figures process, were reported in [180,181].

## 9. Breath-Figure Self-Assembly and Manufacturing of Membranes

Honeycomb polymer films manufactured by breath-figure self-assembly are not directly suitable for membrane applications for two reasons, namely: (a) pores obtained with this process are too large (see Figure 2); (b) pores are not through-pores across the films [182]. However, recent studies have demonstrated that casting a very thin film of polymer solution on ice allows for the preparation of a thin polymer membrane with through-pores, formed by the breath-figures method; the membrane was then transferred onto a porous substrate to form a thin selective layer for microfiltration [183]. Highly ordered porous membranes of cellulose triacetate prepared successfully on ice substrates using breath figure method were reported in [184]. Wan et al. reported manufacturing membranes with breath-figures-inspired honeycomb patterns transferred onto a dense electrospun nanofiber mesh. The alternate approach to manufacturing membranes with the breath-figures technique was discussed in [1], where multilayer interpenetrating porous structures were reported. A similar strategy involving the use of SiO_2_, TiO_2_, Co and Cd nanoparticles for manufacturing membranes demonstrating hierarchical porous structures with nanometrical pores (with dimensions of 2–50 nm) was reported in [185]. Layer-by-layer deposition of carbon nanotubes onto the honeycomb membrane surface, arising from the breath-figures process, allowed for manufacturing voltage-activated membranes demonstrating potential as humidity sensors and microclimate regulators [186]. Breath-figure templating was successfully involved in the preparation of low-resistance microfiltration membranes having a uniform size of pore opening using polysulfone (a regular membrane polymer) [187]. A highly permeable brominated poly(phenylene oxide) microfiltration membrane with binary porous structures, fabricated by a combination of the breath-figure and colloidal crystal template methods, was recently reported in [188]. We conclude that a combination of breath-figures assembly, giving rise to microscaled porous polymer films, with other techniques enabling the formation of nanopores connected to micro-scaled ones has potential in the manufacturing of membranes [183,184,185,186,187,188]. Another useful application of the method is related to the manufacturing of so-called breathing cathodes for fuel cells [189,190].

## 10. Conclusions

Breath-figure self-assembly enables the manufacturing of micro- and sub-micro-scaled porous reliefs demonstrating potential for manufacturing membranes, functionalized templates, sensors, optical and bio-engineering devices, and water-oil and size-selective separation processes [16]. Breath-figure-inspired topographies may be obtained with thermoplastic [14,15,16,37,38,41,44,45,46,61,62] and thermosetting [50,51,52] polymers. High-performance engineering polymers, such as polyimide [53], polyetherimide [54], and polysulfone [45,55], were successfully used for breath-figure self-assembly. 2D and 3D hierarchical structures possessing a range of scales from micrometers to nanometers were reported [14,35,150]. The nature of the large-scale (~10 μm) patterning, attributed by different authors to various kinds of hydrodynamic instabilities, remains disputable [95,96,97,98,117,118].

Breath-figure self-assembly is a robust, single-stage process; however, it involves almost all events inherent to interface science, namely evaporation of a solvent, condensation of water droplets, instabilities developed in the evaporated polymer solution, delayed coalescence of closely packed droplets forming the capillary cluster, and their interaction [16,125,126,127,128,129,130,131]. Thus, the process of breath-figure self-assembly may be understood only within the broader context of surface science [31,32,33,34].

Several basic questions related to the physical and chemical mechanisms of breath-figure self-assembly remain obscure; even the impact of the polymer architecture on the resulting pattern is unclear and calls for future investigations. In this situation the qualitative macroscopic approach to the analysis of the process becomes preferable. Such an analysis is proposed in this review. The estimation of dimensionless numbers describing breath-figure self-assembly, namely the Bond (*Bo*), capillary (*Ca*), and Reynolds (*Re*) numbers, provides evidence that the effects due to gravity, inertia, and viscosity are negligible, at least at the first stage of evaporation of a polymer solution, and that the process is mainly driven by interfacial phenomena. The viscosity of a polymer layer contributes to the stabilization of the pores’ radii.

The hierarchy of spatial and temporal scales inherent to breath-figure self-assembly is elucidated. The characteristic spatial scales of patterns range from dozens of micrometers to nanometers. The temporal scales of the process vary from microseconds to seconds. Analysis of the hierarchy of temporal scales demonstrates that the condensed water droplet almost immediately comes to thermal equilibrium, whereas the polymer solution layer is far from thermal equilibrium. It is also shown that the behavior of a single droplet is quasi-static, whereas the dynamics and thermodynamics of large-scale cells are essentially at non-equilibrium. The topological aspect of self-assembly is considered [119].

Some novel applications and trends of future investigations in the field of breath-figure self-assembly (including a cell-selective surfaces) are envisaged [175,176,177,178,179,180,181]. The use of breath-figures for manufacturing membranes and breathing cathodes was addressed [182,183,184,185,186,187,188,189,190].

## References

[1] Gugliuzza A., Aceto M.C., Macedonio F., Drioli E. (2008). Water droplets as template for next generation self-assembled poly-(etheretherketone) with Cardo membranes. J. Phys. Chem. B.

[2] Ulbricht M. (2006). Advanced functional polymer membranes. Polymer.

[3] Schechter A., Savinell R.F. (2002). Imidazole and 1-methyl imidazole in phosphoric acid doped polybenzimidazole, electrolyte for fuel cells. Solid State Ionics.

[4] Gugliuzza A., Perrotta M.L., Drioli E. (2016). Controlled bulk properties of composite polymeric solutions for extensive structural order of honeycomb polysulfone membranes. Membranes.

[5] Yang J.-S., Swager T.M. (1998). Fluorescent porous polymer films as TNT chemosensors: Electronic and structural effects. J. Am. Chem. Soc..

[6] Li Y.T., Cunin F., Link J.R., Gao T., Betts R.T., Reiver S.H., Chin V. (2003). Polymer replicas of photonic porous silicon for sensing and drug delivery applications. Science.

[7] Rezwan R., Chen Q.Z., Blaker J.J., Boccaccini A.R. (2006). Biodegradable and bioactive porous polymer/inorganic composite scaffolds for bone tissue engineering. Biomaterials.

[8] Lin A.S.P., Barrows T.H., Cartmell S.H., Guldberg R.E. (2003). Microarchitectural and mechanical characterization of oriented porous polymer scaffolds. Biomaterials.

[9] Li H., Zhao X., Wu P., Zhang S., Geng B. (2016). Facile preparation of superhydrophobic and superoleophilic porous polymer membranes for oil/water separation from a polyarylester polydimethylsiloxane block copolymer. J. Mater. Sci..

[10] Cheng B., Lib Z., Li Q., Ju J., Kang W., Naebe M. (2017). Development of smart poly(vinylidene fluoride)-graft-poly(acrylic acid) tree-like nanofiber membrane for pH-responsive oil/water separation. J. Membr. Sci..

[11] Bormashenko E., Balter S., Bormashenko Y., Aurbach D. (2012). Honeycomb structures obtained with breath figures self-assembly allow water/oil separation. Colloids Surf. A.

[12] Liu C., Yang J., Tang Y., Yin L., Tang H., Li C. (2015). Versatile fabrication of the magnetic polymer-based graphene foam and applications for oil–water separation. Colloids Surf. A.

[13] Wan L.-S., Li J.W., Ke B.-B., Xu Z.-K. (2012). Ordered microporous membranes templated by breath figures for size-selective separation. J. Am. Chem. Soc..

[14] Muñoz-Bonilla A., Fernández-García M., Hernández J.R. (2014). Towards hierarchically ordered functional porous polymeric surfaces prepared by the breath figures approach. Prog. Polym. Sci..

[15] Bunz U.H.F. (2006). Breath figures as a dynamic templating method for polymers and nanomaterials. Adv. Mater..

[16] Zhang A., Bai H., Li L. (2015). Breath Figure: A Nature-inspired preparation method for ordered porous films. Chem. Rev..

[17] Aitken J. (1911). Breath figures. Nature.

[18] Rayleigh L. (1911). Breath figures. Nature.

[19] Rayleigh L. (1912). Breath figures. Nature.

[20] Briscoe B., Galvin K. (1991). The effect of surface fog on the transmittance of light. J. Sol. Energy.

[21] Beysens D. (1995). The formation of dew. Atmos. Res..

[22] Beysens D., Knobler C.M. (1986). Growth of breath figures. Phys. Rev. Lett..

[23] Marcos-Martin M., Beysens D., Bouchaud J.P., Godrèche C., Yekutieli I. (1995). Self-diffusion and ‘visited’ surface in the droplet condensation problem (breath figures). Phys. A.

[24] Knobler C.M., Beysens D. (1988). Growth of breath figures on fluid surfaces. Europhys. Lett..

[25] Steyer A., Guenoun P., Beysens D., Knobler C.M. (1990). Two-dimensional ordering during droplet growth on a liquid surface. Phys. Rev. B.

[26] Widawski G., Rawiso B., Francois B. (1994). Self-organized honeycomb morphology of star-polymer polystyrene films. Nature.

[27] Francois B., Pitois O., Francois J. (1995). Polymer films with a self- organized honeycomb morphology. Adv. Mater..

[28] Pitois O., Francois B. (1999). Formation of ordered micro-porous membranes. Eur. Phys. J. B.

[29] Pitois O., François B. (1999). Crystallization of condensation droplets on a liquid surface. Colloid Polym. Sci..

[30] François B., Ederlé Y., Mathis C. (1999). Honeycomb membranes made from C_60_(PS)_6_. Synth. Met..

[31] Adamson A.W., Gast A.P. (1990). Physical Chemistry of Surfaces.

[32] Erbil H.Y. (2006). Surface Chemistry of Solid and Liquid Interfaces.

[33] De Gennes P.-G., Brochard-Wyart F., Quéré D. (2003). Capillarity and Wetting Phenomena.

[34] Bormashenko E. (2013). Wetting of Real Surfaces.

[35] Srinivasarao M., Collings D., Philips A., Patel S. (2001). Three-dimensionally ordered array of air bubbles in a polymer film. Science.

[36] Song L., Bly R.K., Wilson J.N., Bakbak S., Park J.O., Srinivasarao N., Bunz U.H.F. (2004). Facile microstructuring of organic semiconducting polymers by the breath figure method: Hexagonally ordered bubble arrays in rigid rod-polymers. Adv. Mater..

[37] Bolognesi A., Mercogliano C., Yunus S., Civardi M., Comoretto D., Turturro A. (2005). Self-organization of polystyrenes into ordered microstructured films and their replication by soft lithography. Langmuir.

[38] Zhu L.-W., Ou Y., Wan L.-S., Xu Z.K. (2014). Polystyrenes with hydrophilic end groups: Synthesis, characterization, and effects on the self-assembly of breath figure arrays. J. Phys. Chem. B.

[39] Amirkhani M., Berger N., Abdelmohsen M., Zocholl F., Gonçalves M.R., Marti O. (2011). The effect of different stabilizers on the formation of self-assembled porous film via the breath-figure technique. J. Polym. Sci. B.

[40] Peng J., Han Y., Yang Y., Li B. (2004). The influencing factors on the macroporous formation in polymer films by water droplet templating. Polymer.

[41] Ferrari E., Fabbri P., Pilati F. (2011). Solvent and substrate contributions to the formation of breath figure patterns in polystyrene films. Langmuir.

[42] Liu C., Gao C., Yan D. (2007). Honeycomb-patterned photoluminescent films fabricated by self-assembly of hyperbranched polymers. Angew. Chem..

[43] Jenekhe S.A., Chen X.L. (1999). Self-Assembly of ordered microporous materials from rod-coil block copolymers. Science.

[44] Matsuyama H., Ohga K., Maki T., Teramoto M. (2004). The Effect of polymer molecular weight on the structure of a honeycomb patterned thin film prepared by solvent evaporation. J. Chem. Eng. Jpn..

[45] Xu Y., Zhu B., Xu Yo. (2005). A study on formation of regular honeycomb pattern in polysulfone film. Polymer.

[46] Bormashenko E., Pogreb R., Stanevsky O., Bormashenko Y., Gendelman O. (2005). Formation of honeycomb patterns in evaporated polymer solutions: Influence of the molecular weight. Mater. Lett..

[47] Li Z., Ma X., Kong Q., Zang D., Guan X., Ren X. (2016). Static and dynamic hydrophobic properties of honeycomb structured films via breath figure method. J. Phys. Chem. C.

[48] Govor L.V., Bashmakov I.A., Kiebooms R., Dykonov V., Parisi J. (2001). Self-organized networks based on conjugated polymers. Adv. Mater..

[49] Deepak V.D., Asha S.K. (2008). Random and AB diblock copolymers of tricyclodecanemethanol urethane methacrylate with styrene: Synthesis and morphology characterization. J. Polym. Sci. A.

[50] Erdogan B., Song L., Wilson J.N., Park J.O., Srinivasarao M., Bunz U.H.F. (2004). Permanent bubble arrays from a cross-linked poly(para-phenyleneethynylene): Picoliter holes without microfabrication. J. Am. Chem. Soc..

[51] Karikari A.S., Williams A.R., Heisey C.L., Rawlett A.M., Lon T.E. (2006). Porous thin films based on photo-cross-linked star-shaped Poly(d,l-lactide)s. Langmuir.

[52] Zhu L.-W., Yang W., Wan L.-S., Xu Z.-K. (2014). Synthesis of core cross-linked star polystyrene with functional end groups and self-assemblies templated by breath figures. Polym. Chem..

[53] Yabu H., Tanaka M., Ijiro K., Shimomura M. (2003). Preparation of honeycomb-patterned polyimide films by self-organization. Langmuir.

[54] Bormashenko E., Schechter A., Stanevsky O., Stein T., Balter S., Musin A., Bormashenko Y., Pogreb R., Barkay Z., Aurbach D. (2008). Free-standing, thermostable, micrometer-scale honeycomb polymer films and their properties. Macromol. Mater. Eng..

[55] Bormashenko E., Balter S., Malkin A., Aurbach D. (2014). Polysulfone membranes demonstrating asymmetric diode-like water permeability and their applications. Macromol. Mater. Eng..

[56] Gong J., Xu B., Tao X. (2017). Breath figure micromolding approach for regulating the microstructures of polymeric films for triboelectric nanogenerators. ACS Appl. Mater. Interfaces.

[57] Sharma V., Song L., Jones R.L., Barrow M.S., Williams P.R., Srinivasarao M. (2010). Effect of solvent choice on breath-figure-templated assembly of “holey” polymer films. Europhys. Lett..

[58] Bormashenko E., Pogreb R., Stanevsky O., Bormashenko Y., Tamir S., Cohen R., Nunberg M., Gaisin V.-Z., Gorelik M., Gendelman O. (2005). Mesoscopic and submicroscopic patterning in thin polymer films: Impact of the solvent. Mater. Lett..

[59] Battenbo H., Cobley R.J., Wilks S.P. (2011). A quantitative study of the formation of breath figure templated polymer materials. Soft Matter.

[60] Karthaus O., Maruyama N., Cieren X., Shimomura M., Hasegawa H., Hashimoto T. (2000). Water-assisted formation of micrometer-size honeycomb patterns of polymers. Langmuir.

[61] Bormashenko E., Balter S., Aurbach D. (2012). On the Nature of the breath figures self-assembly in evaporated polymer solutions: Revisiting physical factors governing the patterning. Macromol. Chem. Phys..

[62] Bormashenko E., Malkin A., Musin A., Bormashenko Y., Whyman G., Litvak N., Barkay Z., Machavariani V. (2008). Mesoscopic patterning in evaporated polymer solutions: Poly(ethylene glycol) and room-temperature-vulcanized polyorganosilanes/-siloxanes promote formation of honeycomb structures. Macromol. Chem. Phys..

[63] Bormashenko E. (2008). Correct values of Rayleigh and Marangoni numbers for liquid layers deposited on thin substrates. Ind. Eng. Chem. Res..

[64] Hernández-Guerrero M., Stenzel M.H. (2012). Honeycomb structured polymer films via breath figures. Polym. Chem..

[65] Nurmawati B.M.H., Vetrichelvan M., Valiyaveettil S. (2006). Morphological investigations of self-assembled films from a pyridine -incorporated poly (p-phenylene). J. Porous Mater..

[66] Maruyama N., Koito T., Nishida J., Sawadaishi T., Cieren X., Ijiro K., Karthaus O., Shimomura M. (1998). Mesoscopic patterns of molecular aggregates on solid substrates. Thin Solid Films.

[67] Stenzel M.H., Barner-Kowollik C., Davis T.P. (2006). Formation of honeycomb-structured, porous films via breath figures with different polymer architectures. J. Polym. Sci. A.

[68] Kabuto T., Hashimoto Y., Karthaus O. (2007). Thermally stable and solvent resistant mesoporous honeycomb films from a crosslinkable polymer. Adv. Funct. Mater..

[69] Sun W., Ji J., Shen J. (2008). Rings of nanoparticle-decorated honeycomb-structured polymeric film: The Combination of Pickering emulsions and capillary flow in the breath figures method. Langmuir.

[70] Lakshmi V., Raju A., Resmi V.G., Pancrecious J.K., Rajan T.P.D., Pavithran C. (2016). Amino-functionalized breath-figure cavities in polystyrene-alumina hybrid films: Effect of particle concentration and dispersion. Phys. Chem. Chem. Phys..

[71] Wang L.-P., Yin K.-Y., Li G., Liu Q., Deng A.-X., Ma H.-Y. (2016). Rhodamine B-loaded star polystyrenes and their luminescent honeycomb-patterned porous films. React. Funct. Polym..

[72] Madej W., Budkowski A., Raczkowska J., Rysz J. (2008). Breath figures in polymer and polymer blend films spin-coated in dry and humid ambience. Langmuir.

[73] Park M.S., Kim J.K. (2004). Breath figure patterns prepared by spin coating in a dry environment. Langmuir.

[74] Park M.S., Kim J.K. (2005). Broad-band antireflection coating at near-Infrared wavelengths by a breath figure. Langmuir.

[75] Park M.S., Joo W., Kim J.K. (2006). Porous structures of polymer films prepared by spin coating with mixed solvents under humid condition. Langmuir.

[76] Muñoz-Bonilla A., Ibarboure E., Papon E., Rodriguez-Hernandez J. (2009). Self-organized hierarchical structures in polymer surfaces: Self-assembled nanostructures within breath figures. Langmuir.

[77] Arora J.S., Cremaldi J.C., Hollerana M.K., Ponnusamya T., Sunkaraa B., He J., Pesika N.S., John V.T. (2016). Hierarchical patterning of hydrogels by replica molding of impregnated breath figures leads to superoleophobicity. Nanoscale.

[78] Galeotti F., Trespidi F., Pasini M. (2016). Breath figure-assisted fabrication of nanostructured coating on silicon surface and evaluation of its antireflection power. J. Nanomater..

[79] Yabu H., Shimomura M. (2005). Single-step fabrication of transparent superhydrophobic porous polymer films. Chem. Mater..

[80] Yang H., Jiang P. (2010). Self-cleaning diffractive macroporous films by doctor blade coating. Langmuir.

[81] Yang H., Jiang P. (2010). Large-scale colloidal self-assembly by doctor blade coating. Langmuir.

[82] Mansouri J., Yapit E., Chen V. (2013). Polysulfone filtration membranes with isoporous structures prepared by a combination of dip-coating and breath figure approach. J. Membr. Sci..

[83] Van-Tien Bui V.-T., Thuy L.T., Tran Q.C., Nguyen V.-T., Dao V.-D., Choi J.S., Choi H.-S. (2017). Ordered honeycomb biocompatible polymer films via a one-step solution-immersion phase separation used as a scaffold for cell cultures. Chem. Eng. J..

[84] Bera S., Pal M., Sarkar S., Jana S. (2017). Hierarchically structured macro with nested mesoporous zinc Indium Oxide conducting film. ACS Appl. Mater. Interfaces.

[85] Schmelzer J.W.P., Schmelzer J. (2000). Reconciling Gibbs and van der Waals: A new approach to nucleation theory. J. Chem. Phys..

[86] Landau L.D., Lifshitz E.M. (1969). Course of Theoretical Physics Vol 5: Statistical Physics.

[87] Saunders A.T., Dickson J.L., Shah P.S., Lee M.Y., Lim R.T., Johnston K.P., Korgel B.A. (2006). Breath figure templated self-assembly of porous diblock copolymer films. Phys. Rev. E.

[88] Adamson A.W., Gast A.P. (1997). Physical Chemistry of Surfaces.

[89] Lothe J., Pound G.M. (1962). Reconsiderations of nucleation theory. J. Chem. Phys..

[90] Binder R., Stauffer D. (1976). Statistical theory of nucleation, condensation and coagulation. Adv. Phys..

[91] Zeldovich Y.B. (1942). On the theory of formation of new phase. Cavitation. J. Exp. Theor. Phys. USSR.

[92] Sigsbee R.A., Zettlemoyer A.C. (1969). Vapor to condensed-phase heterogeneous nucleation. Nucleation.

[93] Böker A., Lin Y., Chiapperini K., Horowitz R., Thompson N., Carreon V., Xu T., Abetz C., Skaff H., Dinsmore A.D. (2004). Hierarchical nanoparticle assemblies formed by decorating breath figures. Nat. Mater..

[94] Escalé P., Rubatat L., Billon L., Save M. (2012). Recent advances in honeycomb-structured porous polymer films prepared via breath figures. Eur. Polym. J..

[95] Bormashenko E., Pogreb R., Musin A., Stanevsky O., Bormashenko Y., Whyman G., Gendelman O., Barkay Z. (2006). Self-assembly in evaporated polymer solutions: Influence of the solution concentration. J. Colloid Interface Sci..

[96] Bormashenko E., Pogreb R., Stanevsky O., Bormashenko Y., Stein T., Gengelman O. (2005). Mesoscopic patterning in evaporated polymer solutions: New experimental data and physical Mechanisms. Langmuir.

[97] Bormashenko E., Whyman G., Pogreb R., Stanevsky O., Hakham-Itzhaq M., Gendelman O. (2007). Self-assembly in evaporated polymer solutions: Patterning on two scales. Isr. J. Chem..

[98] Nie1 Z., Kumacheva E. (2008). Patterning surfaces with functional polymers. Nat. Mater..

[99] Tokuhisa H., Tsukamoto S., Morita S., Ise S., Tomita M., Shirakawa N. (2017). Fabrication of micro-textured surfaces for a high hydrophobicity by evaporative patterning using screen mesh templates. Appl. Surf. Sci..

[100] Pototsky A., Bestehorn M., Merkt D., Thiele U. (2005). Morphology changes in the evolution of liquid two-layer films. J. Chem. Phys..

[101] Pototsky A., Bestehorn M., Merkt D., Thiele U. (2004). Alternative pathways of dewetting for a thin liquid two-layer film. Phys. Rev. E.

[102] Müller-Buschbaum P., Bauer E., Wunnicke O., Stamm M. (2005). The control of thin film morphology by the interplay of dewetting, phase separation and microphase separation. J. Phys. Condens. Matter.

[103] Colinet P., Legros J.C., Velarde M.G. (2001). Nonlinear Dynamics of Surface-Tension-Driven Instabilities.

[104] Nepomnyashchy A.A., Velarde M.G., Colinet P. (2002). Interfacial Phenomena and Convection.

[105] Reichenbach J., Linde H. (1981). Linear perturbation analysis of surface-tension-driven convection at a plane interface (Marangoni instability). J. Colloid Interface Sci..

[106] Linde H., Velarde M.G., Wierschem A., Waldhelm W., Loeschke K., Rednikov A.Y. (1997). Interfacial wave motions due to Marangoni instability. J. Colloid Interface Sci..

[107] Oron A., Nepomnyashchy A.A. (2004). Long-wavelength thermocapillary instability with the Soret effect. Phys. Rev. E.

[108] Regnier V.C., Dauby P.C., Lebon G. (2000). Linear and nonlinear Rayleigh-Bénard-Marangoni instability with surface deformations. Phys. Fluids.

[109] Zhang N., Chao D.F. (1999). Mechanisms of convection instability in thin liquid layers induced by evaporation. Int. Commun. Heat Mass Transf..

[110] Münch A., Please C.P., Wagner B. (2011). Spin coating of an evaporating polymer solution. Phys. Fluids.

[111] Mitov Z., Kumacheva E. (1998). Convection-induced patterns in phase-separating polymeric fluids. Phys. Rev. Lett..

[112] Minařík A., Smolka P., Minařík M., Mráček A., Rajnohová E., Minaříková M., Gřundělová L., Foglarová M., Velebný V. (2017). A special instrument for the defined modification of polymer properties in solutions and polymer layers. Measurement.

[113] Wrzecionko E., Minařík A., Smolka P., Minařík M., Humpolíček P., Rejmontová P., Mráček A., Minaříková M., Gřundělová L. (2017). Variations of polymer porous surface structures via the time-sequenced dosing of mixed solvents. ACS Appl. Mater. Interfaces.

[114] Fowler P.D., Ruscher C., McGraw J.D., Forrest J.A., Dalnoki-Veress K. (2016). Controlling Marangoni-induced instabilities in spin-cast polymer films: How to prepare uniform films. Eur. Phys. J. E.

[115] Bormashenko E., Balter S., Pogreb R., Bormashenko Y., Gendelman O., Aurbach D. (2010). On the mechanism of patterning in rapidly evaporated polymer solutions: Is temperature-gradient-driven Marangoni instability responsible for the large-scale patterning?. J. Colloid Interface Sci..

[116] Grigoriev R. (2002). Control of evaporatively driven instabilities of thin liquid films. Phys. Fluids.

[117] De Gennes P.G. (2001). Instabilities during the evaporation of a film: Non-glassy polymer + volatile solvent. Eur. Phys. J. E.

[118] De Gennes P.G. (2002). Solvent evaporation of spin cast films: “Crust” effects. Eur. Phys. J. E.

[119] Bormashenko E. (2015). Surface instabilities and patterning at liquid/vapor interfaces: Exemplifications of the “hairy ball theorem”. Colloid Interface Sci. Commun..

[120] Eisenberg M., Guy R. (1979). A proof of the hairy ball theorem. Am. Math. Mon..

[121] Bormashenko E., Aurbach D., Whyman G., Stein T., Bormashenko Y., Pogreb R. (2008). On the role of the Plateau borders in the pattern formation occurring in thin evaporated polymer layers. Colloid Surf. A.

[122] Park S.H., Xia Y. (1998). Macroporous membranes with highly ordered and three-dimensionally interconnected spherical pores. Adv. Mater..

[123] Zhang Y., Wang C. (2007). Micropatterning of proteins on 3D porous polymer film fabricated by using the breath-figure method. Adv. Mater..

[124] Dong R., Yan J., Ma H., Fang Y., Hao J. (2011). Dimensional architecture of Ferrocenyl-based oligomer honeycomb-patterned Films: From monolayer to multilayer. Langmuir.

[125] Kralchevsky P.A., Danov K.D., Denkov N.D., Birdi K.S. (2003). Chemical physics of colloid systems and interfaces. Surface and Colloidal Chemistry.

[126] Kralchevsky P.A., Nagayama K. (1994). Capillary forces between colloidal particles. Langmuir.

[127] Kralchevsky P.A., Nagayama K. (2000). Capillary interactions between particles bound to interfaces, liquid films and biomembranes. Adv. Colloid Interface Sci..

[128] Kralchevsky P.A., Paunov V.N., Ivanov I.B., Nagayama K. (1992). Capillary meniscus interactions between colloidal particles attached to a liquid-fluid interface. J. Colloid Interface Sci..

[129] Bragg L., Nye J.F. (1947). A dynamical model of a crystal structure. Proc. R. Soc. Lond. A.

[130] Lomer W.M. (1949). The forces between floating bubbles and a quantitative study of the Bragg “Bubble Model” of a crystal. Math. Proc. Camb. Philos. Soc..

[131] Peiranski P. (1980). Two-Dimensional interfacial colloidal crystals. Phys. Rev. Lett..

[132] Dong R., Ma H., Yan J., Fang Y., Hao J. (2011). Tunable morphology of 2D honeycomb-patterned films and the hydrophobicity of a Ferrocenyl-based oligomer. Chem. Eur. J..

[133] Dou Y., Jin M., Zhou G., Shui L. (2015). Breath figure method for construction of honeycomb films. Membranes.

[134] Tadmor R. (2004). Line energy and the relation between advancing, receding and Young contact angles. Langmuir.

[135] Xia Y., Gates B., Yin Y., Sun Y., Birdy K.S. (2003). Self-Assembly of Monodispersed Spherical Colloids into Complex Structures. Surface and Colloid Chemistry.

[136] Yin Y., Xia Y. (2001). Self-assembly of monodispersed colloidal spheres into complex aggregates with well-defined sized, shapes and structures. Adv. Mater..

[137] De León A.S., Malhotra S., Molina M., Haag R., Calderón M., Rodríguez-Hernández J., Muñoz-Bonilla A. (2015). Dendritic amphiphiles as additives for honeycomb-like patterned surfaces by breath figures: Role of the molecular characteristics on the pore morphology. J. Colloid Interface Sci..

[138] Wu C.-H., Ting W.-H., Lai Y.-W., Dai S.A., Su W.-C., Tung S.-H., Jeng R.J. (2016). Tailored honeycomb-like polymeric films based on amphiphilic poly(urea/malonamide) dendrons. RSC Adv..

[139] Fedorets A.A. (2005). On the mechanism of non-coalescence in a drop cluster. JETP Lett..

[140] Fedorets A.A., Dombrovsky L.A., Smirnov A.M. (2015). The use of infrared self-emission measurements to retrieve surface temperature of levitating water droplets. Infrared Phys. Technol..

[141] Fedorets A.A., Frenkel M., Shulzinger E., Dombrovsky L.A., Bormashenko E., Nosonovsky M. (2017). Self-assembled levitating clusters of water droplets: Pattern formation and stability. Sci. Rep..

[142] Eggers J., Lister J.R., Stone H.A. (1999). Coalescence of liquid drops. J. Fluid Mech..

[143] Aarts D.G.A.L., Lekkerkerker H.N.W., Guo H., Wegdam G.H., Bonn D. (2005). Hydrodynamics of droplet coalescence. Phys. Rev. Lett..

[144] Karpitschka S., Riegler H. (2014). Sharp transition between coalescence and non-coalescence of sessile drops. J. Fluid Mech..

[145] Karpitschka S., Riegler H. (2010). Quantitative experimental study on the transition between fast and delayed coalescence of sessile droplets with different but completely miscible liquid. Langmuir.

[146] Karpitschka S., Riegler H. (2012). Noncoalescence of sessile drops from different but miscible liquids: Hydrodynamic analysis of the twin drop contour as a self-stabilizing traveling wave. Phys. Rev. Lett..

[147] Dell’Aversana P., Banavar J.R., Koplik J. (1996). Suppression of coalescence by shear and temperature gradients. Phys. Fluids.

[148] Neitzel G.P., Dell’Aversana P. (2002). Noncoalescence and nonwetting behavior of liquids. Annu. Rev. Fluid Mech..

[149] Deegan R.D., Bakajin O., Dupont T.F., Huber G., Nagel S.R., Witten T.A. (1997). Capillary flow as the cause of ring stains from dried liquid drops. Nature.

[150] Deegan R.D., Bakajin O., Dupont T.F., Huber G., Nagel S.R., Witten T.A. (2000). Contact line deposits in an evaporating drop. Phys. Rev. E.

[151] Hu H., Larson R.G. (2006). Marangoni effect reverses coffee-ring depositions. J. Phys. Chem. B.

[152] Hu H., Larson R.G. (2005). Analysis of the Effects of Marangoni stresses on the microflow in an evaporating sessile droplet. Langmuir.

[153] Ma H., Hao J. (2010). Evaporation-induced ordered honeycomb structures of gold nanoparticles at the air/water interface. Chem. Eur. J..

[154] Li J., Peng J., Huang W.H., Wu Y., Fu J., Cong Y., Xue L.J., Han Y.C. (2005). Ordered honeycomb-structured gold nanoparticle films with changeable pore morphology: From circle to ellipse. Langmuir.

[155] Saito Y., Shimomura M., Yabu H. (2014). Breath figures of nanoscale bricks: A universal method for creating hierarchic porous materials from inorganic nanoparticles stabilized with mussel-inspired copolymers. Macromol. Rapid Commun..

[156] Saunders A.E., Shah P.S., Sigman M.B., Hanrath T., Hwang H.S., Lim K.T., Johnston K.P., Korgel B.A. (2004). Inverse opal nanocrystal superlattice films. Nano Lett..

[157] Escalé P., Save M., Billon L., Ruokolainen J., Rubatat L. (2016). When block copolymer self-assembly in hierarchically ordered honeycomb films depicts the breath figure process. Soft Matter.

[158] Roe B.P. (2001). Probability and Statistics in Experimental Physics.

[159] Kumar V.S., Kumaran V. (2005). Voronoi cell volume distribution and configurational entropy of hard-spheres. J. Chem. Phys..

[160] Barthélemy M. (2011). Spatial networks. Phys. Rep..

[161] Limaye A.V., Narhe R.D., Dhote A.M., Ogale S.B. (1996). Evidence for convective effects in breath figure formation on volatile fluid surfaces. Phys. Rev. Lett..

[162] Bormashenko E., Musin A., Whyman G., Barkay Z., Zinigrad M. (2013). Revisiting the fine structure of the triple line. Langmuir.

[163] Yamazaki H., Ito K., Yabu H., Shimomura M. (2014). Formation and control of line defects caused by tectonics of water droplet arrays during self-organized honeycomb-patterned polymer film formation. Soft Matter.

[164] Marmur A., Mittal K.L. (2009). A guide to the equilibrium contact angles maze. Contact Angle Wettability and Adhesion.

[165] Zhou Y., Huang J., Sun W., Ju Y., Yang P., Ding L., Chen Z.-R., Kornfield J.A. (2017). Fabrication of active surfaces with metastable microgel layers formed during breath figure templating. ACS Appl. Mater. Interfaces.

[166] Male U., Shina B.K., Huh D.S. (2017). Coupling of breath figure method with interfacial polymerization: Bottom-surface functionalized honeycomb-patterned porous films. Polymer.

[167] Bormashenko E., Bormashenko Y., Pogreb R., Stanevsky O. (2006). Micrometrically scaled textured metallic hydrophobic interfaces validate the Cassie-Baxter wetting hypothesis. J. Colloid Interface Sci..

[168] Yabu H., Takebayashi M., Tanaka M., Shimomura M. (2005). Superhydrophobic and lipophobic Properties of self-organized honeycomb and pincushion structures. Langmuir.

[169] Cassie A.B.D., Baxter S. (1944). Wettability of porous surfaces. Trans. Faraday Soc..

[170] Cassie A.B.D. (1948). Contact angles. Discuss. Faraday Soc..

[171] Nosonovsky N., Bhushan B. (2009). Superhydrophobic surfaces and emerging applications: Non-adhesion, energy, green engineering. Curr. Opin. Colloid Interface Sci..

[172] Nosonovsky N., Bhushan B. (2008). Biologically inspired surfaces: Broadening the scope of roughness. Adv. Funct. Mater..

[173] Whyman G., Bormashenko E. (2011). How to make the Cassie wetting state stable?. Langmuir.

[174] Arinstein A., Burman M., Gendelman O., Zussman E. (2007). Effect of supramolecular structure on polymer nanofibre elasticity. Nat. Nanotechnol..

[175] Martínez-Campos E., Elzein T., Bejjani A., García-Granda M.J., Santos-Coquillat A., Ramos V., Muñoz-Bonilla A., Rodríguez-Hernández J. (2016). Toward cell selective surfaces: Cell adhesion and proliferation on breath figures with antifouling surface chemistry. ACS Appl. Mater. Interfaces.

[176] Kawano T., Sato M., Yabu H., Shimomura M. (2014). Honeycomb-shaped surface topography induces differentiation of human mesenchymal stem cells (hMSCs): Uniform porous polymer scaffolds prepared by the breath figure technique. Biomater. Sci..

[177] Gong J., Xu B., Tao X., Li L. (2016). Binary breath figures for straightforward and controllable self-assembly of microspherical caps. Phys. Chem. Chem. Phys..

[178] Abbaspour M., Pourabbas B., Azimi M. (2017). Solid-state supercapacitor based on breath figured polymethyl methacrylate deposited by graphene: The effect of electrode surface. J. Mater. Sci. Mater. Electron..

[179] Bormashenko E., Pogreb R., Balter S., Aurbach D. (2013). Electrically controlled membranes exploiting Cassie-Wenzel wetting transitions. Sci. Rep..

[180] Saito Y., Yabu H. (2015). Bio-inspired low frictional surfaces having micro-dimple arrays prepared with honeycomb patterned porous films as wet etching masks. Langmuir.

[181] Kamei J., Abe H., Yabu H. (2017). Biomimetic bubble-repellent tubes: Microdimple arrays enhance repellency of bubbles inside of tubes. Langmuir.

[182] Wang D.-M., Lai J.-H. (2013). Recent advances in preparation and morphology control of polymeric membranes formed by nonsolvent induced phase separation. Curr. Opin. Chem. Eng..

[183] Cong H.L., Wang J.K., Yu B., Tang J. (2012). Preparation of a highly permeable ordered porous microfiltration membrane of brominated poly(phenylene oxide) on an ice substrate by the breath figure method. Soft Matter.

[184] Yu B., Cong H., Li Z., Yuan H., Peng Q., Chi ., Yang Sh., Yang R., Wickramasinghe S.R., Tang J. (2015). Polymer Sci. B. Fabrication of highly ordered porous membranes of cellulose triacetate on ice substrates using breath figure method. J. Polym. Sci. B.

[185] Sakatani Y., Boissière C., Grosso D., Nicole L., Soler-Illia G.J.A.A., Sanchez C. (2008). Coupling nanobuilding block and breath figures approaches for the designed construction of hierarchically templated porous materials and membranes. Chem. Mater..

[186] Gugliuzza A., Pingitore V., Drioli E. (2016). Relationships between structure and electrical sensing of breathable membranes. Mater. Today Proc..

[187] Tripathi B.K., Pande P. (2014). Breath figure templating for fabrication of polysulfone microporous membranes with highly ordered monodispersed porosity. J. Membr. Sci..

[188] Yuan H., Yu B., Cong H., Peng Q., Yang Z., Luo Y., Chi M. (2016). A highly permeable brominated poly(phenylene oxide) (BPPO) microfiltration membrane with binary porous structures was fabricated by combination of the breath figure and colloidal crystal template methods. J. Colloid Interface Sci..

[189] Li J., Zhang N., Ni D., Sun K. (2011). Preparation of honeycomb porous solid oxide fuel cell cathodes by breath figures method. Int. J. Hydrog. Energy.

[190] Li J., Zhang N., Ni D., Sun K. (2011). Preparation of honeycomb porous La_0.6_Sr_0.4_Co_0.2_Fe_0.8_O_3-δ_Gd_0.2_Ce_0.8_O_2-δ_ composite cathodes by breath figures method for solid oxide fuel cells. Appl. Surf. Sci..

